# Exploring the Physical Activity Counselling for Patients With Rheumatoid Arthritis—Perceptions of Nurses and Physiotherapists

**DOI:** 10.1002/msc.70053

**Published:** 2025-01-10

**Authors:** Katja Lehtomäki, Iina Ryhtä, Jaana Peltonen, Minna Stolt

**Affiliations:** ^1^ Department of Nursing Science University of Turku Turku Finland; ^2^ Tyks General medicine, Western area of responsibility The Wellbeing Services County of Southwest Finland Turku Finland; ^3^ Faculty of Health and Welfare Satakunta University of Applied Science Pori Finland; ^4^ Research and Development Unit The Wellbeing Services County of Satakunta Pori Finland

**Keywords:** competence, counselling, interview, nurse, physical activity, physiotherapy, rheumatoid arthritis

## Abstract

**Background:**

Physical activity is beneficial for patients with rheumatoid arthritis (RA). However, little is known about how healthcare professionals counsel and support patients with RA to be physically active.

**Aim:**

This study aims to explore nurses' and physiotherapists' perceptions of delivering physical activity counselling for patients with RA during routine follow‐up appointments.

**Method:**

A mixed‐methods convergent parallel design was employed, using survey and interview data from nurses and physiotherapists (*n* = 9) at a rheumatology outpatient clinic. Statistical and inductive content analyses were conducted. COREQ guidelines were followed.

**Findings:**

Nurses and physiotherapists perceived physical activity counselling to be central in the overall RA care. They considered it partly challenging as delivering effective counselling requires understanding the specific characteristics of RA and tailoring advice to the individual patient's needs. In general, they focused on motivating patients to be physically active by demonstrating the benefits of physical activity. However, they also reported a lack of awareness regarding the content discussed during each other's appointments, highlighting issues with information sharing between professionals.

**Conclusion:**

Physical activity counselling is multidimensional, constitutes part of professional competence and requires extensive knowledge of RA and physical activity recommendations. Future interventions are needed to enhance nurses' and physiotherapists' competence in delivering physical activity counselling.

## 1 Introduction

Physical activity is an essential part of everyday life, particularly for individuals with long‐term health problems, such as rheumatoid arthritis (RA). RA is an autoimmune disease characterised by joint inflammation and functional impairments. More than 17 million people worldwide, primarily women, are affected by RA, and its prevalence is expected to rise (GBD 2021). Physical activity and functional exercises are beneficial and safe, improving functional ability, joint range of motion and quality of life (Katz, Andonian, and Huffman 2020).

The care of patients with RA is carried out through multiprofessional collaboration, where registered nurses and physiotherapists play a particularly central role (NICE 2020). These professionals are vital for patient education and providing support for physical activity. Providing such counselling requires not only an understanding of RA but also theoretical knowledge of individually tailored exercises and their benefits (Edelaar et al. 2020). Physical activity counselling belongs to health promotion (WHO 1986) and is part of patient consultation where the aim is to support or change physical activity behaviour through primary or secondary prevention and, for example, exercise advice (Estabrooks, Glasgow, and Dzewaltowski 2003). In this study, physical activity counselling refers to guidance and instruction implemented by a health care professional to exercise safely and adequately, and to finding suitable forms of exercise and physical activity (Stoutenberg et al. 2018). Despite the importance of physical activity counselling, little is known about how healthcare professionals (registered nurses and physiotherapists) deliver physical activity counselling to support patients with RA in maintaining an active lifestyle.

The World Health Organisation (WHO) aims to reduce global inactivity in adults by 15% by the year 2030 (WHO 2018a). Inactivity remains a significant issue, with about 30% of adults globally classified as inactive (WHO 2018b), and rates are even higher among those with long‐term health conditions such as RA (Summers et al. 2019). Addressing this requires targeted physical activity counselling and promotion of active lifestyles that meet recommended activity levels (WHO 2020; Rheumatoid Arthritis. Current Care Guidelines 2017). Patients with RA are recommended to follow the international guidelines of aerobic physical activity where adults should perform at week‐level at least 150–300 min of moderate‐intensity activities or at least 75–150 min of vigorous intensity activities (WHO 2020).

Physical activity counselling is an important component of consultations regarding long‐term health conditions, aiming to change physical activity behaviours through primary or secondary prevention (Gwinnutt et al. 2021). Competence among healthcare professionals is critical, with knowledge of RA forming the foundation for effective planning and delivery of physical activity interventions (Thomas et al. 2023). Evidence suggests that lifestyle physical activity interventions not only increase physical activity levels but also reduce disease activity in RA patients (Brady et al. 2023).

Research on nurses' and physiotherapists' perceptions of physical activity counselling is scarce. However, their roles are central in RA care (van Hell‐Cromwijk et al. 2021). Physiotherapists often focus on recommending and guiding specific exercises for musculoskeletal conditions, while nurses play an important and active role in general physical activity counselling and prioritise interdisciplinary collaboration (van Hell‐Cromwijk et al. 2021; O'Brien et al. 2020). Nevertheless, the use of behaviour change strategies remains limited, and environmental factors, such as lack of community‐based opportunities for physical activity, also pose challenges (Zhu et al. 2021). Additionally, consultation time and the complexity of patients' healthcare needs further restrict the scope of physical activity counselling (Albert et al. 2020).

The methods used to deliver physical activity counselling vary widely (Wattanapisit, Wattanapisit, and Wongsiri 2021). While some patients report satisfaction with the counselling they receive (Thomsen et al. 2024), others feel it is inadequate or absent (Freid, Ogdie, and Baker 2020). Approaches range from personalised exercise programmes to digital platforms (Katz, Andonian, and Huffman 2020). Motivational interviewing and the transtheoretical model are frequently used to promote physical activity (Söderlund et al. 2018; Kleis et al. 2021). Personalised exercise plans are particularly effective in addressing individual patient needs (Weijers, Rongen‐van Dartel, and van Riel 2018). When caring for patients with long‐term musculoskeletal health conditions, such as RA, individually tailored support and counselling are crucial to improve the patient's current life situation (Sweeney et al. 2023). Evidence also supports the use of nurse‐led interventions that combine disease management with physical activity for RA patients (Lopatina et al. 2021).

Given that patients with RA often face challenges maintaining physical activity, particularly in the early years of the disease (Gwinnutt et al. 2021), it is essential to provide individualised counselling that aligns with their specific physical health needs. However, relatively little is known about how nurses and physiotherapists provide this support in practice.

## 2 Aim

This study aims to explore nurses' and physiotherapists' perceptions of delivering physical activity counselling for patients with RA during routine follow‐up appointments. In doing so, it seeks to provide insights to enhance physical activity counselling and improve rehabilitation and care quality for patients with RA. The following research questions guided the study:

— How do nurses and physiotherapists deliver physical activity counselling for patients with RA?
— What are nurses' and physiotherapists' perceptions of delivering physical activity counselling for patients with RA?

## 3 Methods

### 3.1 Design

A mixed‐methods convergent parallel design was applied in this study (Creswell and Plano Clark 2018). The study was reported in accordance with the COnsolidated criteria for REporting Qualitative research (COREQ) guidelines (Tong, Sainsbury, and Craig 2007).

### 3.2 Sampling and Data Collection

Purposive sampling was used to recruit informants from one Finnish hospital district, which includes four hospitals with rheumatology outpatient clinics. An information letter about the study was distributed by named contact persons or head nurses of the rheumatology clinics to all registered nurses and physiotherapists working there. Potential participants were eligible if they were registered nurses or physiotherapists, worked at the rheumatology clinic and cared for patients with RA on a daily basis. Those willing to participate informed the contact persons, who then provided the researcher (K.L.) with their contact details. The researcher subsequently arranged the time and place for the interviews.

Data were collected through surveys and individual interviews by the first author, who was a nurse with a bachelor's degree in nursing science and long work experience in nursing. First, participants completed a structured survey questionnaire on the content and frequency of physical activity counselling provided to patients with RA. Frequency was measured using Likert scale response options (1 = seldom, 2 = occasionally, 3 = often). Second, individual face‐to‐face interviews were conducted and audio recordings were recorded. These interviews elaborated on participants' views and perceptions regarding delivering physical activity counselling by employing structured questions and an interview guide. The guide, informed by prior research (e.g., NICE 2020; Edelaar et al. 2020; Freid, Ogdie, and Baker 2020), covered two main themes: the content of physical activity counselling during routine appointments with RA patients and the competence requirements for delivering such counselling. The first interview was considered as a pilot test to ensure the functionality of the survey and interview questions. No modifications were made after the pilot.

Background information was also collected, including the participants' profession (registered nurse or physiotherapist), years of experience in health care and years of experience in caring for patients with RA. Data collection occurred between May and July 2022. The interviews were conducted either remotely (using Zoom) or face to face in a hospital meeting room. Any other people were not present in the interviews besides the participants and researcher. The interviews lasted 35–60 min.

### 3.3 Data Analysis

Data were analysed using descriptive statistical methods and inductive content analysis. Quantitative data were processed using SPSS (SPSS Statistics for Windows, Version 28.0. Armonk, NY: IBM Corp.) for descriptive statistical analysis (percentages and frequencies). Qualitative data from the interviews were transcribed verbatim, resulting in 63 A4 pages (Times New Roman, size 12, spacing 1.0) and analysed using inductive content analysis, focusing on manifest content (Graneheim and Lundman 2004). The unit of analysis was sentences or phrases containing words relating to physical activity counselling. These units addressed specific research questions on how physical counselling is conducted in outpatient clinics, the content areas covered and nurses' and physiotherapists' competencies in this area. Meaning units were condensed, abstracted and labelled with codes (*n* = 173). Codes sharing common characteristics were grouped into subcategories (*n* = 25), which were further organised into categories (*n* = 8). Finally, these categories were grouped into three main categories based on their content. Quantitative and qualitative data were analysed concurrently but separately by two researchers (K.L., M.S.). No software was used to manage the data.

### 3.4 Ethical Considerations

The study followed good scientific practice (ALLEA 2023). According to legislation in Finland, the nature of this study did not require ethical review (TENK 2019). Permission to conduct the study was obtained from the organisation in February 2022. An information letter stating the purpose of the study, data collection procedures, anonymity and confidentiality in reporting and the possibility to withdraw was delivered to every participant. Each participant gave written informed consent to participate in the study.

## 4 Results

### 4.1 Description of the Participants

The participants (*n* = 9) included registered nurses (*n* = 5) and physiotherapists (*n* = 4) working in rheumatology outpatient clinics. Their average healthcare working experience was 19 years (range: 4–33, standard deviation: 9.0). Their average experience caring for patients with RA was 10 years (range: 0.25–20, standard deviation: 6.6).

#### 4.1.1 Physical Activity Counselling Delivered by Nurses and Physiotherapists for Patients With RA

Physical activity counselling provided to patients with RA by nurses and physiotherapists was diverse (Table 1). Both groups frequently discussed patients' physical activity habits and the benefits of being physically active. The participants found such discussions straightforward as patients with RA were generally receptive to advice on improving their physical activity. A patient‐centred approach was prioritised, with healthcare professionals striving to empower patients.

**TABLE 1:** Nurses' and physiotherapists' reported forms and frequency of physical activity counselling for patients with RA.

	Seldom		Occasionally		Often	
	Nurses	Physiotherapists	Nurses	Physiotherapists	Nurses	Physiotherapists
Discussion of patient's physical activity habit					X	X
Discussion of benefits regarding physical activity					X	X
Discussion of current care guideline in physical activity promotion	X		X		X	X
Delivery of information about:						
Improving one's aerobic fitness	X					X
Improving muscle strength	X			X		X
Maintaining the range of motion in joints	X			X		X
Demonstration of functional exercises of:						
Muscle strength	X			X		
Joint range of motion	X		X	X		
Description of alternative forms of physical activity (such as swimming, water running)			X		X	X
Patient education about:						
Prevention of injuries	X		X	X	X	X
Self‐management of minor injuries			X	X	X	X
Discussion about negative preconceptions regarding physical activity (e.g., fear of movement)			X		X	X
Intention to find ways to motivate the patient to promote physical activity					X	X
Counselling to group‐based exercise	X		X	X	X	

Some role‐specific differences were identified. Physiotherapists discussed endurance and muscle strength exercises, maintaining the range of motion and suitable exercises and activities more than nurses. Meanwhile, nurses provided general physical activity counselling, focusing on healthy lifestyles and activities of daily living. Nurses felt their competence in providing detailed guidance on specific exercises was limited, considering physiotherapists to have greater expertise in this area. In turn, physiotherapists perceived their role as delivering in‐depth tailored counselling and designing detailed exercise programmes for patients. They recognised nurses' contributions to general physical activity counselling and overall RA care. Physiotherapists used demonstrations to guide patients in performing exercises correctly and offered alternative ways to stay active, tailoring recommendations to individual needs and resources. They emphasised adapting information to align with patients' capabilities and circumstances.

Motivating patients to be physically active was a central goal of physical activity counselling. Motivation was enhanced by discussing the general benefits of physical activity as well as its specific role in managing RA. Participants frequently referred patients to third‐sector activities, such as exercise sessions and events organised by regional rheumatism associations. These collective activities were seen as promoting not only physical activity but also social peer support for patients with RA.

#### 4.1.2 Nurses' and Physiotherapists' Perceptions of Delivering Physical Activity Counselling for Patients With RA

**FIGURE 1:**
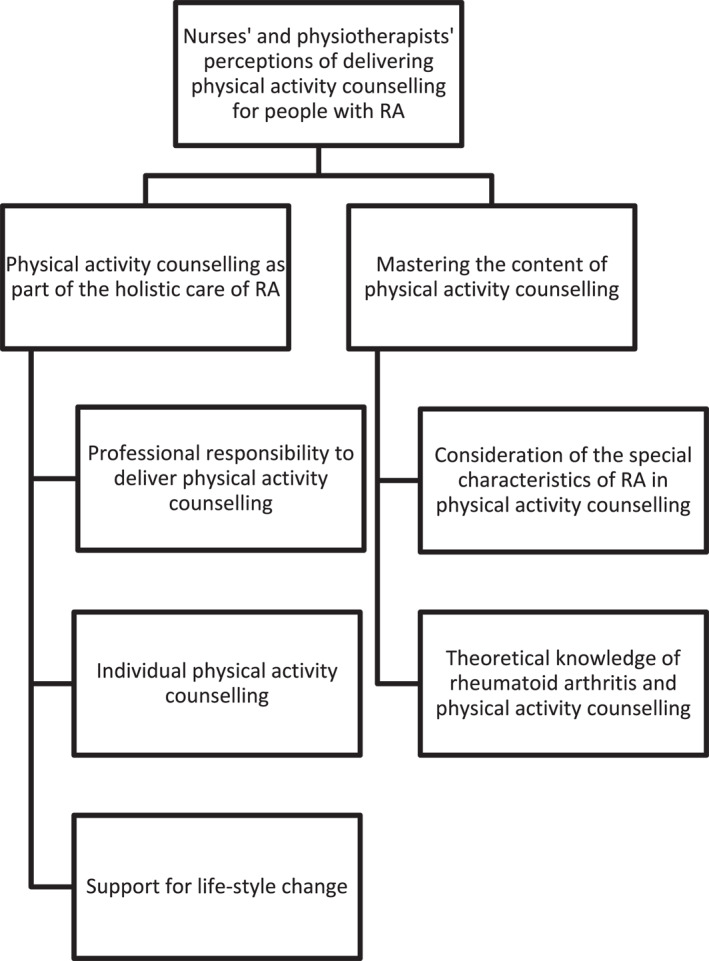
Nurses' and physiotherapists' perceptions of delivering physical activity counselling for people with rheumatoid arthritis.

The perceptions of nurses and physiotherapists regarding the delivery of physical activity counselling for patients with RA were categorised into two main categories: physical activity counselling as a part of holistic care for RA and mastering the content of physical activity counselling (Figure 1). Physical activity counselling was viewed as an integral component of holistic RA care and divided into three subcategories: professional responsibility to deliver counselling, individualised counselling and support for lifestyle change.

Providing physical activity counselling was seen as a cornerstone of effective care, delivered collaboratively by multidisciplinary teams. Nurses were perceived as playing a pivotal role, particularly as they are often the first point of contact for newly diagnosed RA patients. During initial appointments, nurses typically provided general information about RA under a long‐term condition and guidance on medication management. Due to time constraints, comprehensive patient education sessions (lasting 30–60 min) were often scheduled separately.

Nurses identified their role in physical activity counselling as general, often integrated with advice on broader lifestyle changes, such as diet, sleep and overall health. They emphasised physiotherapists' role in delivering detailed information about physical activity promotion.

I try to follow the content of national Current Care Guideline of rheumatoid arthritis and counsel patients as well as I can. I also trust physiotherapists have the competence to provide tailored physical activity counselling and collaborate effectively.

(Nurse ID5)

Physiotherapists typically provided physical activity counselling 2–4 months after diagnosis, during appointments lasting 60–90 minutes. These appointments focused more specifically on physical activity. While nurses acknowledged that counselling on physical activity could be overlooked in favour of other priorities (e.g., medication management), physiotherapists viewed it as central to their role.

In the acute phase of the disease, I guide patients to engage in light activity that is suitable within the limits of pain and discomfort. But in the remission state, physical activity can be increased progressively.

(Physiotherapist ID2)

Participants reported a lack of awareness regarding the content discussed during each other's appointments, highlighting issues with information sharing between professionals. To overcome these problems, they expressed a willingness to improve collaboration, although no concrete initiatives had been implemented. Additionally, they frequently used educational leaflets, written materials and digital health platforms provided by national organisations to support their counselling. Patients were also encouraged to explore resources from local municipalities and patient organisations.

Individual physical activity counselling was considered important. Participants highlighted the importance of identifying patients' individual needs, preferences and resources. They emphasised understanding each patient's unique circumstances through medical records and conversations, using this information to guide their recommendations. The respondents also emphasised the importance of understanding the nature of RA as a chronic condition and recognising how patients' physical activity levels may fluctuate throughout the disease course.

The content of physical activity counselling is tailored according to patient’s current health status and possible health concerns and needs.

(Nurse ID2)

Motivating patients to increase their daily activity levels gradually was a key focus. Participants underscored that all physical activities that are performed in the course of daily activities are useful, along with structured activities, such as group‐based exercises. They noted that patients have varying attitudes towards the benefits of physical activity counselling. Some are eager to be physically active, while others prefer a more passive lifestyle. Participants emphasised the need to understand patients' individual experiences with physical activity and have the ability to motivate patients to be more physically active to mitigate the symptoms of RA. In particular, they perceived a need for more information on how to motivate and support older patients and those with no previous experience of regular physical exercise.

I try to motivate the patients to be physically active on daily basis and consider that all little steps, like walking to the post box or short walking trips, are all good for your health.

(Nurse ID1)

Participants identified challenges in motivation due to the fluctuating nature of RA symptoms, including pain, joint stiffness, fatigue and medication side effects. Motivational interviewing and physical tests (e.g., grip strength and timed up‐and‐go tests) were seen as effective strategies to engage patients by allowing them to compare their results with population norms.

Support and counselling related to lifestyle changes were viewed as especially important. Participants emphasised the need to understand patients well and adapt counselling to their individual capabilities. Encouragement and the development of trusting relationships with patients are considered central to successful counselling. However, participants noted that as healthcare appointments became less frequent over time, many patients lacked adequate support to sustain physical activity. Participants emphasised the importance of ongoing engagement, tailored guidance and recognising patients' unique resources in making sustainable lifestyle changes.

It would be excellent, if patient organisations could organise supervised exercise groups or other easy to reach activities. These could help patients find interesting hobbies.

(Physiotherapists ID3)

Mastering the content of physical activity counselling was divided into two subcategories: consideration of the special characteristics of RA in physical activity counselling, and theoretical knowledge of RA and physical activity counselling.

Delivering effective physical activity counselling for patients with RA requires understanding the unique characteristics of the disease and possessing theoretical knowledge of counselling strategies. Overall, competence in this area was seen as crucial. In particular, participants underscored the importance of recognising the fluctuating nature of RA symptoms, which alternate between periods of increased disease activity and relative remission. Healthcare professionals need to assess the patient's disease status and adapt their physical activity counselling accordingly. Understanding the impact of pharmacological treatments and how medication affects physical activity was also deemed essential. Tailoring physical activities to support patients' functional status, particularly during remission periods, was highlighted.

I need to know how rheumatoid arthritis changes body functions. Knowing that makes it easier to plan exercise programmes.

(Physiotherapist ID1)

The ability to modify and tailor counselling to fit the specific needs of RA patients was considered vital. Participants highlighted the importance of familiarity with national care guidelines and evidence‐based recommendations for physical activity counselling. They acknowledged that patients with RA might not engage in physical activities in the same way as healthy individuals. Knowing joint‐friendly activities, such as aquatic exercises, and offering alternative ways to remain active were emphasised as key components of effective counselling.

To improve patients' adherence to physical activity, I need to know effective patient education methods—not only provide written materials to patients but also try to motivate them to be active every day.

(Physiotherapist ID4)

Participants identified self‐directed learning and motivation to seek information as important for staying updated on counselling methods. However, limited opportunities for in‐service training and continuous education on physical activity counselling for RA were seen as barriers to professional development. Multiprofessional collaboration and learning from peers were recognised as additional avenues for enhancing competence.

Healthcare professionals caring for patients with rheumatoid arthritis need to have basic knowledge of rheumatoid arthritis and must also stay up to date and deepen their knowledge.

(Physiotherapist ID2)

## 5 Discussion

Physical activity counselling for patients with RA requires extensive knowledge of the disease and effective counselling methods. This study highlights the importance of both general and specialised competence among healthcare professionals to improve physical activity outcomes for RA patients.

Physical activity counselling is a critical component of holistic care delivered by a multiprofessional team. Nurses and physiotherapists were found to collaborate effectively, with nurses focusing on basic‐level information and physiotherapists providing tailored counselling based on individual patient needs. These findings align with previous studies (van Hell‐Cromwijk et al. 2021; Thomsen et al. 2024). However, the studies identified a need for deeper competence in physical activity counselling among both nurses and physiotherapists, particularly those specialising in RA. Enhanced training and resources are essential to ensure that healthcare professionals can deliver high‐quality, patient‐centred care.

The views of nurses and physiotherapists on guiding patients with RA in physical activity highlight the importance of professional competence. Counselling as part of the overall treatment of RA involves implementing physical activity counselling, offering personalised mobility guidance and supporting the lifestyle changes. Mastery of the content of physical activity counselling is fundamental to providing professional care.

Nurses primarily described their physical activity counselling as foundational and broad. However, they expressed confidence in physiotherapists' ability to provide individually tailored guidance. This aligns with previous studies, which have shown seamless collaboration between nurses and physiotherapists in RA care (van Hell‐Cromwijk et al. 2021; O'Brien et al. 2020). Nonetheless, some participants noted delay or gaps in information flow, underscoring the need for robust communication structures and interprofessional knowledge sharing. Clear roles and understanding of professional expertise in physical activity counselling can improve care quality and patient outcomes (Reeves et al. 2017).

The results of this study support earlier findings indicating that motivational interviewing improved physical activity (Zhu et al. 2024). However, evidence on the long‐term effectiveness of motivational interviewing remains inconclusive (Zhu et al. 2024). Therefore, modern methods for supporting physical activity must be explored and implemented. Nurses and physiotherapists reported using written materials, such as leaflets, brochures, or links to websites, but there were no mentions of mobile applications (Bearne et al. 2020). Such emerging tools could provide patients with continuous guidance, education and encouragement to stay active (Fedkov et al. 2022).

Physical activity counselling remains a relevant topic in healthcare (Rausch Osthoff et al. 2018). With the global prevalence of RA increasing (GBD 2021) and many patients failing to meet recommended daily activity levels, efforts must focus on improving the competence of nurses and physiotherapists in RA‐specific counselling. The findings of the study can guide the planning of targeted educational initiatives and resource allocation to enhance the treatment of RA.

### 5.1 Methodological Considerations

A mixed‐methods research design (Regnault, Willgoss, and Barbic 2017) was used to gain a comprehensive understanding of nurses' and physiotherapists' perceptions of delivering physical activity counselling to RA patients. Participants first completed a structured questionnaire about the content and frequency of their counselling practices, followed by interviews to gain deeper insights into their delivery methods, content and collaboration. Saturation was reached in interviews as repetition of main features of physical activity counselling was identified.

Data analysis was conducted independently by two researchers (K.L., M.S.), with peer debriefing and member checking ensuring the reliability of interpretations. Peer debriefing involved ongoing discussions within the research team during the analysis process, while member checking was performed by an experienced physiotherapist not involved in data collection but with substantial experience in RA physiotherapy. Transparent reporting with authentic expressions was used to reflect participants' voices accurately.

Purposive sampling ensured the inclusion of participants with expertise in RA physical activity counselling from a university hospital. However, as the data were collected at a single university hospital in Finland, the applicability of these results to other contexts may require further validation due to differences in healthcare systems and services.

## 6 Conclusions

Nurses and physiotherapists play active and important roles in physical activity counselling for patients with RA during routine consultations. Nurses primarily focus on general physical activity counselling, whereas physiotherapists provide individually tailored physical activity counselling. Knowledge of the specific characteristics of RA and its impact on physical activity is crucial, and thus both nurses and physiotherapists could benefit from in‐service training focused on delivering physical activity counselling. The findings can help inform the design and testing of rehabilitative interventions aiming to improve physical activity among RA patients.

## Author Contributions

K.L. and M.S. participated in the design of the study. K.L. and M.S. conducted the data collection and analysis. Interpretation of results was contributed by all authors. K.L., I.R., and J.P. drafted the manuscript and M.S. supervised the process and participated in editing the manuscript. All authors have read and approved the final version of the manuscript.

## Conflicts of Interest

The authors declare no conflicts of interest.

## Data Availability

The authors have nothing to report.

## References

[msc70053-bib-0001] Albert, F. A. , M. J. Crowe , A. E. O. Malau‐Aduli , and B. S. Malau‐Aduli . 2020. “Physical Activity Promotion: A Systematic Review of the Perceptions of Healthcare Professionals.” International Journal of Environmental Research and Public Health 17, no. 12: 4358. 10.3390/ijerph17124358. 32570715 PMC7345303

[msc70053-bib-0002] ALLEA . 2023. The European Code of Conduct for Research Integrity – Revised Edition 2023. Berlin. 10.26356/ECOC.

[msc70053-bib-0003] Bearne, L. M. , M. Sekhon , R. Grainger , et al. 2020. “Smartphone Apps Targeting Physical Activity in People With Rheumatoid Arthritis: Systematic Quality Appraisal and Content Analysis.” JMIR mHealth and uHealth 8, no. 7: e18495. 10.2196/18495. 32706727 PMC7404016

[msc70053-bib-0004] GBD 2021 Rheumatoid Arthritis Collaborators . 2023. “Global, Regional, and National Burden of Rheumatoid Arthritis, 1990–2020, and Projections to 2050: A Systematic Analysis of the Global Burden of Disease Study 2021.” Lancet. Rheumatology 5, no. 10: e594–e610. 10.1016/S2665-9913(23)00211-4. 37795020 PMC10546867

[msc70053-bib-0005] Brady, S. M. , J. J. C. S. Veldhuijzen van Zanten , P. C. Dinas , et al. 2023. “Effects of Lifestyle Physical Activity and Sedentary Behaviour Interventions on Disease Activity and Patient‐ and Clinician‐Important Health Outcomes in Rheumatoid Arthritis: A Systematic Review With Meta‐Analysis.” BMC Rheumatology 7, no. 1: 27. 10.1186/s41927-023-00352-9. 37674187 PMC10481589

[msc70053-bib-0006] Creswell, J. W. , and V. L. Plano Clark . 2018. Designing and Conducting Mixed Methods Research. 3rd. ed. Los Angeles: SAGE Publications, Inc.

[msc70053-bib-0007] Edelaar, L. , E. Nikiphorou , G. E. Fragoulis , et al. 2020. “2019 EULAR Recommendations for the Generic Core Competences of Health Professionals in Rheumatology.” Annals of the Rheumatic Diseases 79, no. 1: 53–60. 10.1136/annrheumdis-2019-215803. 31399400

[msc70053-bib-0008] Estabrooks, P. A. , R. E. Glasgow , and D. A. Dzewaltowski . 2003. “Physical Activity Promotion through Primary Care.” Journal of the American Medical Association 289, no. 22: 2913–2916. 10.1001/jama.289.22.2913. 12799388

[msc70053-bib-0009] Fedkov, D. , A. Berghofen , C. Weiss , et al. 2022. “Efficacy and Safety of a Mobile App Intervention in Patients With Inflammatory Arthritis: A Prospective Pilot Study.” Rheumatology International 42, no. 12: 2177–2190. 10.1007/s00296-022-05175-4. 36112186 PMC9483251

[msc70053-bib-0010] Freid, L. M. , A. Ogdie , and J. F. Baker . 2020. “Physical Activity Patterns in People With Inflammatory Arthritis Indicate They Have Not Received Recommendation‐Based Guidance From Health Care Providers.” ACR Open Rheumatology 2, no. 10: 582–587. 10.1002/acr2.11183. 32985797 PMC7571386

[msc70053-bib-0011] Graneheim, U. H. , and B. Lundman . 2004. “Qualitative Content Analysis in Nursing Research: Concepts, Procedures and Measures to Achieve Trustworthiness.” Nurse Education Today 24, no. 2: 105–112. 10.1016/j.nedt.2003.10.001. 14769454

[msc70053-bib-0012] Gwinnutt, J. M. , H. Alsafar , K. L. Hyrich , et al. 2021. “Do People With Rheumatoid Arthritis Maintain Their Physical Activity Level at Treatment Onset over the First Year of Methotrexate Therapy?” Rheumatology 60, no. 10: 4633–4642. 10.1093/rheumatology/keab060. 33605404 PMC8487269

[msc70053-bib-0013] Katz, P. , B. J. Andonian , and K. M. Huffman . 2020. “Benefits and Promotion of Physical Activity in Rheumatoid Arthritis.” Current Opinion in Rheumatology 32, no. 3: 307–314. 10.1097/BOR.0000000000000696. 32141951

[msc70053-bib-0014] Kleis, R. R. , M. C. Hoch , R. Hogg‐Graham , and J. M. Hoch . 2021. “The Effectiveness of the Transtheoretical Model to Improve Physical Activity in Healthy Adults: A Systematic Review.” Journal of Physical Activity and Health 18, no. 1: 94–108. 10.1123/jpah.2020-0334. 33260143

[msc70053-bib-0015] Lopatina, E. , D. A. Marshall , S. A. Le Clercq , et al. 2021. “Nurse‐Led Care for Stable Patients With Rheumatoid Arthritis: Quality of Care in Routine Practice Compared to the Traditional Rheumatologist‐Led Model.” Rheumatology and Therapy 8, no. 3: 1263–1285. 10.1007/s40744-021-00339-3. 34236650 PMC8380599

[msc70053-bib-0016] NICE . 2020. Rheumatoid Arthritis in Adults: Management. NICE Guideline. https://www.nice.org.uk/guidance/ng100.

[msc70053-bib-0017] O'Brien, M. W. , C. A. Shields , K. L. Campbell , S. J. Crowell , and J. R. Fowles . 2020. “Perceptions and Practices of Providing Physical Activity Counselling and Exercise Prescriptions Among Physiotherapists in Nova Scotia.” Physiotherapy Canada 72, no. 3: 230–238. 10.3138/ptc-2018-0098. 35110791 PMC8781476

[msc70053-bib-0018] Rausch Osthoff, A. K. , K. Niedermann , J. Braun , et al. 2018. “2018 EULAR Recommendations for Physical Activity in People With Inflammatory Arthritis and Osteoarthritis.” Annals of the rheumatic diseases 77, no. 9: 1251–1260. 10.1136/annrheumdis-2018-213585. 29997112

[msc70053-bib-0019] Reeves, S. , F. Pelone , R. Harrison , J. Goldman , and M. Zwarenstein . 2017. “Interprofessional Collaboration to Improve Professional Practice and Healthcare Outcomes.” Cochrane Database of Systematic Reviews 6, no. 6: CD000072. 10.1002/14651858.CD000072.pub3. 28639262 PMC6481564

[msc70053-bib-0020] Regnault, A. , T. Willgoss , and S. Barbic . 2017. “Towards the Use of Mixed Methods Inquiry as Best Practice in Health Outcomes Research.” Journal of Patient‐Reported Outcomes 2, no. 1: 19. 10.1186/s41687-018-0043-8. 29757311 PMC5934918

[msc70053-bib-0021] Rheumatoid Arthritis. Current Care Guidelines . 2017. Working Group Set up by the Finnish Medical Society Duodecim and the Finnish Society for Rheumatology. Helsinki: Finnish Medical Society Duodecim. www.kaypahoito.fi.

[msc70053-bib-0022] Söderlund, P. D. 2018. “Effectiveness of Motivational Interviewing for Improving Physical Activity Self‐Management for Adults With Type 2 Diabetes: A Review.” Chronic Illness 14, no. 1: 54–68. 10.1177/1742395317699449. 29226694

[msc70053-bib-0023] Stoutenberg, M. , K. I. Galaviz , F. Lobelo , et al. 2018. “A Pragmatic Application of the RE‐AIM Framework for Evaluating the Implementation of Physical Activity as a Standard of Care in Health Systems.” Preventing Chronic Disease 15: E54. 10.5888/pcd15.170344. 29752803 PMC5951671

[msc70053-bib-0024] Summers, G. , A. Booth , K. Brooke‐Wavell , T. Barami , and S. Clemes . 2019. “Physical Activity and Sedentary Behavior in Women With Rheumatoid Arthritis: A Comparison of Patients With Low and High Disease Activity and Healthy Controls.” Open Access Rheumatology Research and Reviews 11: 133–142. 10.2147/OARRR.S203511. 31417323 PMC6592056

[msc70053-bib-0025] Sweeney, A. T. , C. A. Flurey , C. S. McCabe , J. C. Robson , P. Richards , and M. Ndosi . 2023. “Nurse‐Led Care for People With Early Rheumatoid Arthritis: Interview Study With Thematic Analysis.” Musculoskeletal Care 21, no. 4: 1651–1661. 10.1002/msc.1844. 37988223

[msc70053-bib-0038] TENK . 2019. The Ethical Principles of Research with Human Participants and Ethical Review in the Human Sciences in Finland. https://tenk.fi/sites/default/files/2021‐01/Ethical_review_in_human_sciences_2020.pdf.

[msc70053-bib-0026] Thomas, R. , A. Berry , C. Swales , and F. Cramp . 2023. “Strategies to Enhance Physical Activity in People With Rheumatoid Arthritis: A Delphi Survey.” Musculoskeletal Care 21, no. 3: 723–732. 10.1002/msc.1745. 36883597

[msc70053-bib-0027] Thomsen, T. , M. Aadahl , M. L. Hetland , and B. A. Esbensen . 2024. “Physical Activity Guidance in the Rheumatology Clinic‐What Matters for Patients With Rheumatoid Arthritis? A Qualitative Study.” Rheumatology International 44, no. 1: 181–189. 10.1007/s00296-023-05466-4. 37787914 PMC10766747

[msc70053-bib-0028] Tong, A. , P. Sainsbury , and J. Craig . 2007. “Consolidated Criteria for Reporting Qualitative Research (COREQ): A 32‐item Checklist for Interviews and Focus Groups.” International Journal for Quality in Health Care 19, no. 6: 349–357. 10.1093/intqhc/mzm042. 17872937

[msc70053-bib-0029] van Hell‐Cromwijk, M. , S. F. Metzelthin , L. Schoonhoven , C. Verstraten , W. Kroeze , and J. M. de Man van Ginkel . 2021. “Nurses' Perceptions of Their Role With Respect to Promoting Physical Activity in Adult Patients: A Systematic Review.” Journal of Clinical Nursing 30, no. 17–18: 2540–2562. 10.1111/jocn.15747. 33899286

[msc70053-bib-0030] Wattanapisit, A. , S. Wattanapisit , and S. Wongsiri . 2021. “Overview of Physical Activity Counseling in Primary Care.” Korean Journal of Family Medicine 42, no. 4: 260–268. 10.4082/kjfm.19.0113. 32429011 PMC8321902

[msc70053-bib-0031] Weijers, J. M. , S. A. A. Rongen‐van Dartel , and P. L. C. M. van Riel . 2018. “Exercise Participation Has Increased in Patients With Rheumatoid Arthritis: A Cross‐Sectional Comparison Between Two Dutch RA Cohorts.” Mediterranean Journal of Rheumatology 29, no. 4: 199–206. 10.31138/mjr.29.4.199. 32185327 PMC7045938

[msc70053-bib-0032] WHO . 1986. Ottawa Charter for Health Promotion, 1986. Regional Office for Europe, World Health Organization. https://iris.who.int/handle/10665/349652.

[msc70053-bib-0033] WHO . 2018a. Global Action Plan on Physical Activity 2018–2030: More Active People for a Healthier World. Geneva: World Health Organization. https://www.who.int/publications/i/item/9789241514187.

[msc70053-bib-0034] WHO . 2018b. Global Levels of Physical Inactivity in Adults: Off Track for 2030. https://iris.who.int/bitstream/handle/10665/378026/9789240096905‐eng.pdf.

[msc70053-bib-0035] WHO . 2020. WHO Guidelines on Physical Activity and Sedentary Behaviour. Geneva: World Health Organization.

[msc70053-bib-0036] Zhu, S. , C. Sherrington , M. Jennings , et al. 2021. “Current Practice of Physical Activity Counselling Within Physiotherapy Usual Care and Influences on Its Use: A Cross‐Sectional Survey.” International Journal of Environmental Research and Public Health 18, no. 9: 4762. 10.3390/ijerph18094762. 33947018 PMC8125383

[msc70053-bib-0037] Zhu, S. , D. Sinha , M. Kirk , et al. 2024. “Effectiveness of Behavioural Interventions With Motivational Interviewing on Physical Activity Outcomes in Adults: Systematic Review and Meta‐Analysis.” BMJ 386: e078713. 10.1136/bmj-2023-078713. 38986547 PMC11234249

